# Meningococcal Antibiotic Resistance: Molecular Characterization of Isolates from Patients with Invasive Meningococcal Disease (IMD) in Greece

**DOI:** 10.3390/antibiotics12071136

**Published:** 2023-06-30

**Authors:** Ioanna Spiliopoulou, Athanasia Xirogianni, Stelmos Simantirakis, Georgina Tzanakaki

**Affiliations:** 1 National Meningitis Reference Laboratory, Department of Public Health Policy, School of Public Health, University of West Attica, 11521 Athens, Greece; i.spiliopoulou@eody.gov.gr (I.S.); axirogianni@uniwa.gr (A.X.); ssimantirakis@uniwa.gr (S.S.); 2 ECDC Fellowship Programme, Public Health Microbiology Path (EUPHEM), European Centre for Disease Prevention and Control (ECDC), 16973 Solna, Sweden; 3 National Public Health Organization (NPHO), Central Public Health Laboratory, 16672 Attica, Greece

**Keywords:** *N. meningitidis*, antimicrobial resistance, *penA* allele, *gyrA* allele, *rpoB* allele

## Abstract

For effective case management and chemoprophylaxis of Invasive Meningococcal Disease (IMD), prompt antibiotic treatment is required. *N. meningitidis* is usually susceptible to antibiotics, but reduced susceptibility to penicillin, ciprofloxacin, and rifampicin is increasing worldwide, jeopardizing patients’ outcome. We assessed, phenotypically and genotypically, the antimicrobial resistance patterns of 192 strains isolated from IMD cases from all over Greece during 2010–2021. Antimicrobial susceptibility to penicillin, rifampicin, and ciprofloxacin was determined using the E-test. All isolates were genotyped by Multilocus Sequence Typing (MLST). *penA*, *rpoB,* and *gyrA* genes were amplified by PCR and sequenced. Of the 192 isolates, 37% (72/192) were penicillin-susceptible/had increased exposure, and 11% (21/192) were penicillin-resistant. Among those, 40 *penA* alleles were identified; *penA1*, *penA27,* and *penA3* were highly associated with susceptibility to penicillin; *penA14*, *penA25,* and *penA22* related to reduced susceptibility to penicillin, while *penA9*, *penA910,* and *penA295* had resistance to penicillin. Two ciprofloxacin-resistant isolates harbored the *gyrA346* allele, while one rifampicin-resistant isolate harbored the *rpoB5* allele. Resistance to ciprofloxacin and rifampicin remains rare. As Greece is one of the countries with high antimicrobial resistance, continued monitoring of antibiotic resistance is important to ensure timely detection of emerging resistance for treatment and prevention guidelines.

## 1. Introduction

Invasive meningococcal disease (IMD), caused by *Neisseria meningitidis,* is a severe, life-threatening illness posing a major worldwide health problem as an important cause of morbidity and mortality. Meningitis, or septicaemia, are the main clinical presentations of IMD. Globally, there are approximately half a million cases of IMD each year [1], with the incidence varying across geographical regions and case fatality rates ranging from 4.1% to 20% [2]. In Europe, an average incidence rate of 0.6 cases per 100,000 people was reported [3], with the highest incidence rates observed in children <1 year old, followed by a second peak amongst adolescents and young adults [3]. Worldwide, the serogroups responsible for the majority of IMD cases are A, B, C, W, X, and Y [1,4], while, in Europe, the most prevalent serogroups are B and C. Although, in recent years, an increase in IMD due to serogroup W has been reported [3]. Multilocus sequence typing (MLST) classifies meningococcal strains into different sequence types (STs), with related STs grouped into lineages termed clonal complexes (ccs) [5].

Due to the severity of the disease, suitable and prompt patient management is of great importance with regards to the administration of antibiotics, even prior to admission to the hospital in order to improve the patient’s outcome. Suspected IMD is treated empirically with third-generation cephalosporins (cefotaxime or ceftriaxone). Upon confirmation, treatment options include cefotaxime, ceftriaxone, penicillin G, or ampicillin. On the other hand, in order to prevent secondary IMD cases, chemoprophylaxis with ciprofloxacin and rifampicin is recommended for close contacts [6].

Although *N. meningitidis* is still susceptible to most antibiotics used for treatment, such as penicillin and third-generation cephalosporins, the emergence and expansion of meningococcal clones resistant to these antibiotics may jeopardize the patient’s outcome. In recent years, there are increasing reports worldwide of either penicillin resistance or reduced susceptibility to penicillin [7,8,9,10,11,12,13,14], directly related to amino acid substitutions (AASs) (F504L, A510V, I515V, H541N, and I566V) in the *penA* gene, which encodes an altered penicillin-binding protein, PBP2, with a reduced affinity for binding penicillin [15].

Furthermore, resistance to antibiotics used for chemoprophylaxis (ciprofloxacin and rifampicin), relatively uncommon in the past, is being reported more frequently worldwide [12,13,16,17,18,19,20,21,22,23]. Resistance to ciprofloxacin is due to mutations in the quinolone resistance-determining region (QRDR) of the *gyrA* gene, encoding DNA gyrase subunit A. Further, enhanced levels of ciprofloxacin resistance can be also observed on mutations in the *parC* gene, enconding DNA topoisomerase IV, subunit A [17]. Recently, ciprofloxacin resistance was reported for the first time in Greece in a migration camp, belonging to the ST-3129 clone [20]. Further, rifampicin resistance due to alterations in the *rpoB* gene encoding the β subunit of RNA polymerase, although uncommon, has been reported among meningococci due to the administration of rifampicin for close contact chemoprophylaxis [21,22,23].

As Greece is among the countries reporting a high percentage of antimicrobial resistance, it is of great importance to monitor the antimicrobial susceptibility of *N. meningitidis* in order to provide evidence for decision-making bodies. The first report on antibiotic susceptibility of *N. meningitidis* isolates from patients and carriers was in early 1990s [24], followed by a recent study on epidemiology of invasive meningococcal disease in Greece during 2006 to 2016 [25], regarding the phenotypic evaluation of antimicrobial resistance patterns given for this period. However, no further information describing the antibiotic susceptibility in relation to the molecular characteristics of the strains causing meningococcal disease in Greece has been provided up to now.

The aim of this study was to identify the *N. meningitidis* antimicrobial resistance patterns, both phenotypically and genotypically, exhibiting either resistance or reduced susceptibility to antibiotics used for treatment (penicillin) or chemoprophylaxis (ciprofloxacin and rifampicin) in strains isolated from patients all over Greece during a 12-year period (2010–2021).

## 2. Results

### 2.1. Serogroup Distribution

Among the 192 isolates, MenB accounted for 84% (162/192)—predominant throughout the study period—followed by MenY (6%; 12/192), MenC (5%; 10/192), MenW (4%; 7/192), and MenX (1%; 1/192).

### 2.2. Clonal Complex Distribution

Among the 192 isolates, 71 STs were found, according to MLST analysis, and they were grouped into 17 ccs. The majority (81%; 155/192) of the isolates (all serogroups included) belonged to the following eight ccs: cc269 (21%; 41/192), cc41/44 (15%; 28/192), cc32 (14%; 27/192), cc213 (8%; 16/192), cc11 (7%; 14/192), cc162 (6%; 11/192), cc23 (5%; 10/192), and cc35 (4%; 8/192). The remaining isolates belonged to less prevalent ccs (*n* = 23) or were not assigned to any cc (*n* = 14). In particular, the majority of the MenB isolates belonged to cc269 (25%; 41/162), cc41/44 (17%; 28/162), cc32 (17%; 27/162), followed by cc213 (10%; 16/162) and cc162 (7%; 11/162). The majority of MenY isolates belonged to cc23 (83%; 10/12), MenC isolates belonged to cc11 (90%; 9/10), while MenW isolates belonged to cc11 (57%; 4/7) and cc22 (29%; 2/7). The single MenX isolate was not assigned to any cc.

### 2.3. Susceptibility to Antibiotics

Of the 192 tested isolates, 99 (52%) were penicillin-susceptible, standard exposure (Pen^S^; MICs 0.006–0.064 mg/L), 72 (37%) were penicillin-susceptible, increased exposure (Pen^I^; MICs 0.094–0.25 mg/L), and 21 (11%) were penicillin-resistant (Pen^R^; MICs 0.38–0.75 mg/L).

Two isolates were ciprofloxacin-resistant (MIC = 0.25 mg/L), while 190 isolates were ciprofloxacin-sensitive (MICs ≤ 0.002–0.016 mg/L). The MIC_50_ for ciprofloxacin was 0.008 mg/L, and the MIC_90_ was 0.012 mg/L. The two ciprofloxacin-resistant isolates were also resistant to penicillin (MIC = 0.5 mg/L).

One isolate was rifampicin-resistant (MIC = 0.38 mg/L), while 191 isolates were rifampicin-sensitive (MICs = 0.003–0.25 mg/L). The MIC_50_ and MIC_90_ were 0.016 mg/L and 0.094 mg/L, respectively (Table 1).

All isolates were sensitive to cefotaxime (MICs = <0.002–0.125 mg/L) and ceftriaxone (MICs = <0.002–0.094 mg/L).

#### 2.3.1. Susceptibility to Penicillin

**Figure 1:**
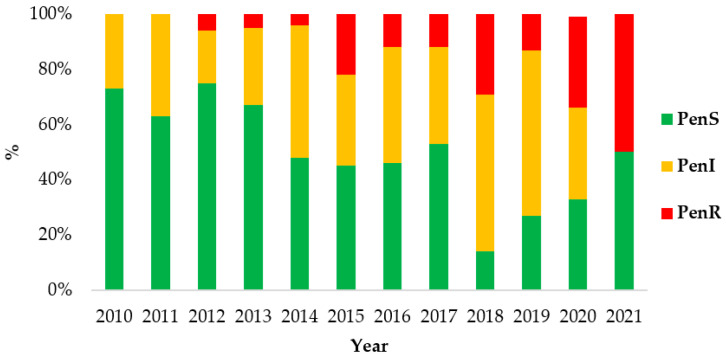
Susceptibility to penicillin among meningococcal isolates by year, Greece, 2010–2021.

Throughout the study period, a reduction in the Pen^S^ isolates was observed with a simultaneous increase in both Pen^I^ and Pen^R^ isolates. The MIC_50_ for penicillin was 0.064 mg/L, and the MIC_90_ was 0.38 mg/L. Although, in 2021, only two isolates were received, and one was penicillin-resistant (Figure 1).

##### Susceptibility to Penicillin in Relation to Serogroups

**Figure 2:**
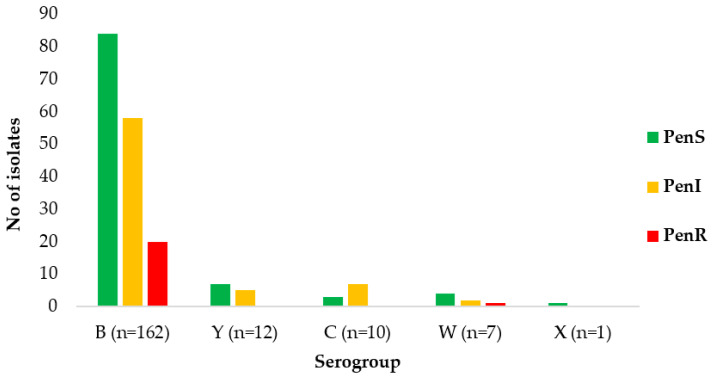
Penicillin susceptible isolates in relation to serogroups, Greece, 2010–2021.

In general, among the 162 MenB isolates, 52% (84/162) were penicillin-susceptible. The proportion of Pen^S^ isolates belonging to MenY, MenC, MenW, and MenX were 58% (7/12), 30% (3/10), 57% (4/7), and 100% (1/1), respectively (Figure 2).

The majority of Pen^R^ isolates belonged to MenB (95%; 20/21), while 5% (1/21) belonged to MenW. A decrease in the proportion of MenB Pen^S^ isolates was observed over the study period, ranging from 79% in 2012 to 11% in 2018 (data not shown). No Pen^R^ isolates were detected among MenY and MenC isolates.

##### Susceptibility to Penicillin in Relation to Clonal Complexes

**Figure 3:**
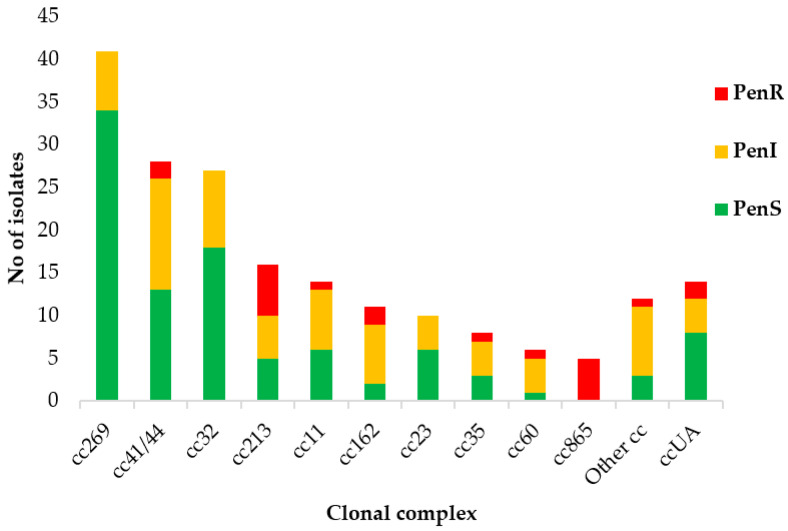
Susceptibility to penicillin in relation to clonal complexes, Greece, 2010–2021.

Overall, the most prevalent clonal complex was cc269 (21%), followed by cc41/44 (15%) (all related to MenB), while the most prevalent clonal complexes among Pen^S^ isolates were cc269, cc32, and cc23. In contrast, cc213 and cc865 were most prevalent among the Pen^R^ isolates (Figure 3).

#### 2.3.2. The Distribution of *penA* Alleles

**Figure 4:**
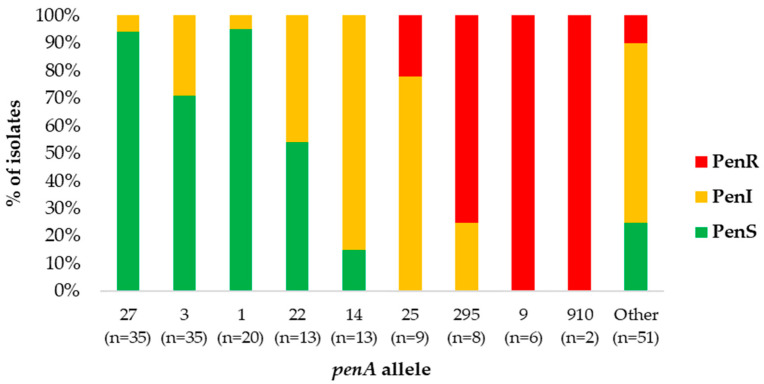
Distribution of *penA* alleles among the meningococcal isolates in relation to penicillin susceptibility, Greece, 2010–2021.

Forty (40) *penA* alleles were identified (Table S1). *penA1*, *penA27,* and *penA3* were highly associated with a susceptibility to penicillin (95%, 94%, and 71%, respectively). Alleles *penA14*, *penA25,* and *penA22* were highly associated with a reduced susceptibility to penicillin (85%, 78%, and 46%, respectively) (MIC values 0.094–0.25 mg/L). In contrast, *penA9*, *penA910,* and *penA295* were highly associated with resistance to penicillin (100% for *penA9* and *penA910* and 75% for *penA295*) (Figure 4).

**Figure 5:**
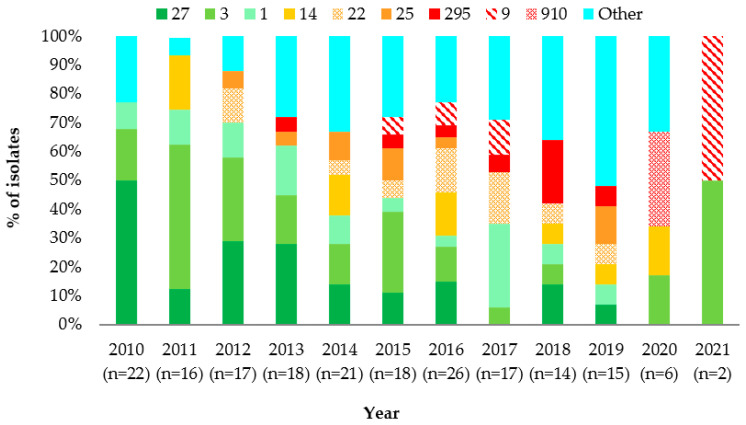
Distribution of *penA* alleles by year during the study, Greece, 2010–2021.

A reduction in the *penA27* allele was observed from 2010 to 2013, following a further reduction from 2014 to 2021, while alleles *penA1* and *penA3* were relatively stable throughout the study period. Similarly, there was not a significant change among the alleles highly associated with a reduced susceptibility to penicillin (*penA14*, *penA22*, and *penA25*) during the study period. In contrast, the alleles highly associated with penicillin resistance (*penA9*, *penA910,* and *penA295*) were detected from 2015 onwards (Figure 5).

Two new *penA* alleles were identified (Table S2). *penA1185,* belonging to ST-269 (cc269), was Pen^I^ (MIC = 0.19 mg/L), while *penA1189,* belonging to ST-13136 (cc60), was Pen^R^ (MIC = 0.75 mg/L). Both *penA* alleles were submitted to the *Neisseria* PubMLST database (http://pubmlst.org/neisseria/) (accessed on 20 February 2023) [26].

#### 2.3.3. Distribution of *penA* Alleles in Relation to Serogroups and Clonal Complexes

Alleles *penA3* and *penA27* were found in MenB isolates and were highly associated with cc32 (74%; 26/35) and cc269 (100%; 35/35), respectively (Table S2). The *penA1* allele was mostly associated with MenB cc41/44 (45%; 9/20), MenY cc11, and MenW cc11 (15%; 3/20, respectively). The *penA25* allele was highly associated with MenB cc162 (89%; 8/9), while *penA22* was highly associated with MenY cc23 (69%; 9/13). In contrast, *penA14* was highly diverse and not related to any specific serogroup or clonal complex. Nine (9) *penA* alleles were identified among the 21 Pen^R^ isolates (Table S2). All possessed five amino acid substitutions (AASs) (F504L, A510V, I515V, H541N, and I566V) in the *penA* gene associated with penicillin resistance and reduced susceptibility [14]. The Pen^R^ isolates that harbored *penA9* allele were mostly associated with MenB cc865 (5/6; 83%) or MenW cc11 (1/6; 17%). The *penA295* allele was identified in all the MenB cc213 Pen^R^ isolates. The *penA910* allele identified in two MenB, ST-3129 (unassigned cc) Pen^R^ isolates were recovered from an outbreak that occurred in a migration camp in the Greek island of Lesbos [19], also exhibiting ciprofloxacin resistance.

#### 2.3.4. Susceptibility to Ciprofloxacin

The majority of the isolates (99%) were sensitive to ciprofloxacin, harboring mainly the alleles *gyrA4* (33%; 63/192), *gyrA12* (25%; 48/192), *gyrA2* (20%; 38/192), and *gyrA3* (11%; 22/192). Further, two new *gyrA* alleles were identified (*gyrA396* and *gyrA397*), both belonging to MenB ST-163 (163cc) and ST-162 (162cc), respectively, and they were submitted to the *Neisseria* PubMLST database (http://pubmlst.org/neisseria/) (accessed on 20 February 2023) [26]. Two strains belonging to MenB and ST-3129 (unassigned cc) that harbored the *gyrA346* allele were resistant to ciprofloxacin (MIC = 0.25 mg/L) and were isolated for the first time in Greece during an outbreak in a migration camp [19]. A T91I mutation within the *gyrA*-QRDR was identified. Both isolates were also resistant to penicillin (MIC = 0.5 mg/L).

#### 2.3.5. Susceptibility to Rifampicin

The majority (99.5%) of the isolates were sensitive to rifampicin. The most frequent alleles were *rpoB4* (33%; 63/192), *rpoB2* (15%; 28/192), *rpoB18,* and *rpoB34* (13%; 24/192 respectively), followed by *rpoB28* (8%; 16/192) and *rpoB5* (5%; 10/192). Four new rifampicin-sensitive *rpoB* alleles were identified; *rpoB281* (MenX, ST-11135, unassigned cc), *rpoB282* (MenB, ST-12882, unassigned cc), *rpoB283* (MenB, ST-12984, cc865), and *rpoB284* (MenB, ST-213, cc213) were submitted to the *Neisseria* PubMLST database (http://pubmlst.org/neisseria/) (accessed on 20 February 2023) [26]. One rifampicin-resistant isolate (MIC = 0.38 mg/L) harbored the *rpoB5* allele and belonged to MenB, ST-12983 (unassigned cc). The strain was isolated from a sporadic case.

## 3. Discussion

The present study describes, for the first time in Greece, the genotypic detection of antibiotic resistance to penicillin, ciprofloxacin, and rifampicin among *N. meningitidis* isolated from IMD cases. In a similar previous Greek study, during the years 1989 to 1991 [24], no resistance to ciprofloxacin and rifampicin was reported. However, nearly half the isolates (48.3%) were reported with reduced susceptibility to penicillin. This percentage was quite high, mainly due to different penicillin breakpoints implemented at that time. In the present study, 52% of isolates were penicillin-susceptible, and standard exposure (Pen^S^) was in line with studies from Italy (55%) [7] and lower than in the UK (63%) [8]. Furthermore, 37% exhibited penicillin susceptibility, increased exposure (Pen^I^) in agreement with studies from UK (34%) [8], and lower than Italy (45%) [7] and Australia (59%) [13]. The present study showed that 11% of isolates were penicillin-resistant, in agreement with data from Australia (13%) [13] and higher than those from the UK (3%) [8], the USA (<0.1%) [9], and Italy (0.7%) [7].

During the 12-year study period, the percentage of Pen^I^ isolates has increased over time, which is consistent with global trends, as multiple countries have reported an increased proportion of Pen^I^ isolates since 2000 [7,10,11,27]. Although there is no evidence to suggest that isolates with reduced susceptibility are associated with treatment failure, the increased Pen^I^ isolates could still pose a concern for individuals with hereditary or acquired complement deficiencies and persons being treated with complement inhibitors, who sometimes receive long-term penicillin prophylaxis [4,28].

In total, 40 *penA* alleles were identified; alleles *penA9*, *penA910,* and *penA295* were highly associated with penicillin resistance in line with other studies, for which *penA9* was also identified in Pen^R^ isolates in studies from the UK [8], Belgium [10], and Japan [12], while the *penA295* allele was also detected among the Pen^R^ isolates in a study from the UK [8]. The *penA910* allele was identified among the Greek Pen^R^ isolates; to our knowledge, this allele has not been reported in Pen^R^ isolates from other studies. Alleles *penA14*, *penA25,* and *penA22* were found to be highly associated with reduced susceptibility to penicillin. Our findings come to an agreement with studies from Italy [7] and the UK [8], where the *penA14* has also been reported in Pen^I^ isolates. The identification of Pen^I^ isolates harboring the *penA25* allele is in agreement with studies from the UK [8] and Italy [7], although we detected it in a higher frequency. The *penA22* allele was identified mostly in Pen^I^ isolates, which is in agreement with the UK study [8]. However this allele has also been identified in Pen^S^ isolates, in agreement with a study from Ireland [27]. Alleles *penA1*, *penA27,* and *penA3* were found to be highly associated with susceptibility to penicillin. The *penA1* allele has been detected in Pen^S^ isolates in studies from the UK [8], Ireland [27], and Brazil [29], although in a higher proportion compared to our study. In contrast, the *penA27* allele has also been identified in Pen^S^ isolates in studies from the UK [8] and Ireland [27], but in a lower proportion compared to our findings. *penA3* was mostly associated with Pen^S^ isolates, which is in line with studies from the UK [8], Ireland [27], and Brazil [29].

The majority of MenB cc269 and cc32 isolates were Pen^S^ and harbored the *penA27* and *penA3* alleles, respectively. The majority of MenB cc162 Pen^I^ isolates were highly associated with the *penA25* allele, in contrast to a previous study where the *penA14* allele has been reported in Pen^I^ MenB cc162 isolates [7]. Half of the MenB cc213 isolates harbored the *penA295* allele and were either Pen^I^ or Pen^R^, in line with a previous study [7]. The majority of the MenY isolates, belonging to cc23 [7,30,31,32,33], harbored the *penA22* allele and were either Pen^S^ or Pen^I^, in contrast to previous studies [7], where the *penA20* allele was identified among the MenY cc23 Pen^I^ isolates. Most of the isolates belonging to the hypervirulent clone cc11, mainly associated with MenC [7,30,31], were Pen^I^, harboring mainly the *penA248* allele, in agreement with a previous study [7]. Although the invasive MenW cc11 lineage has been detected in our study, the majority of isolates were Pen^S^, highly associated with the *penA1* allele, while only one isolate was Pen^R^, harboring the *penA9* allele that has been reported in Pen^R^ strains circulating in several countries [12,34].

Although ciprofloxacin resistance is rare worldwide [7,8,9,12,29,30], a recent study from China has shown that the average ciprofloxacin susceptibility rate across all serogroups was 24.9% [35], and, since 2004, all ciprofloxacin-resistant isolates of the various clonal complexes contained a T91I mutation in the *gyrA* gene, with more genetic diversity for *gyrA* compared with susceptible strains [36]. This causes an ongoing concern regarding the increasing prevalence of ciprofloxacin-resistant *N. meningitidis* globally. In the present study, only two isolates were ciprofloxacin-resistant (MIC = 0.25 mg/L), harboring the *gyrA346* allele with a T91I mutation, belonging to ST-3129 (unassigned cc) and closely related to an isolate from China, indicating that the ciprofloxacin-resistant isolates were imported. Further, 99% of the isolates were sensitive to ciprofloxacin; the *gyrA4* allele was the most prevalent and in agreement with other studies [31].

Almost all isolates were sensitive to rifampicin (99.5%; 191/192). The most frequent allele was *rpoB4*. However, one strain was found resistant and derived from a sporadic case with no evidence of being a close contact of an index case, harboring the *rpoB5* allele. However, no mutations in the *rpoB* gene associated with high rifampicin resistance was found, in contrast to a previous study in Brazil where the allele *rpoB14*, which is associated with high rifampicin resistance, was detected in one isolate [29]. Nonetheless, rifampicin resistance seems to be stable in Greece, as it remains low and in agreement with the previous study [24]. Further analysis is needed to identify the possible mechanisms responsible for the resistance to rifampicin of this particular strain.

Some limitations in our study include that our results reflect only culture confirmed cases due to early antibiotic treatment prior to sampling (40% vs. 60% non-culture confirmed). Furthermore, due to the COVID-19 pandemic, the number of cases and isolates was low during the years 2020–2021 due to the sharp decrease in IMD cases in Greece [37] and worldwide [38]. Nonetheless, this study provides the first insight into the molecular characterization of invasive *N. meningitidis* isolates in Greece as data collected represent the whole country.

From a public health perspective, as Greece has a high percentage of antimicrobial resistance in both the community and hospital settings, monitoring the antimicrobial susceptibility of *N. meningitidis* is of utmost importance. Sustained genomic surveillance of AMR among meningococci is essential to monitor for the emergence and evolution of resistant clones carrying specific resistance genes that could jeopardize the effective national IMD chemoprophylaxis and treatment strategies.

## 4. Materials and Methods

### 4.1. Source of Specimens

A total of 486 IMD cases were confirmed for the period 2010–2021 (average incidence 0.4 per 100,000 population). All samples, cerebrospinal fluid (CSF), and/or blood (depending on the patient’s clinical presentation), as well as isolates, were sent to the National Meningitis Reference Laboratory (NMRL) from hospitals throughout the country for further identification. However, due to early administration of antibiotic treatment prior to sampling, 40% (192/486) of the cases were cultured and confirmed, while 60% (294/486) were confirmed solely by PCR, for which no culture isolates were available. As the present study aims susceptibility testing, we focused on the 192 culture confirmed cases. Patients’ age ranged from <12 months to 88 years old (median age 18 years), while 90 patients were males, and 102 were females.

### 4.2. Identification

Meningococcal isolates were cultured on Chocolate Columbia Agar (OXOID Ltd., Basingstoke, UK) and incubated at 37 °C and 5–10% CO_2_ for 24 h, with a further *N. meningitidis* confirmation by the application of a multiplex PCR, as previously described [39]. Serogroups were determined by a slide agglutination test (Remel Europe Ltd., Dartford, Kent, UK) according to the manufacturer’s instructions.

### 4.3. Antibiotic Susceptibility Testing

E-test was deployed for determining the Minimum Inhibitory Concentration (MIC) for penicillin, rifampicin, and ciprofloxacin by the use of MIC test strip methods (LIOFILCHEM S.r.l, Teramo, Italy) on Mueller-Hinton agar supplemented with 5% sheep blood (OXOID Ltd., Basingstoke, UK) and incubated at 37 °C according to the manufacturer’s instructions. The values were interpreted according to the European Committee on Antimicrobial Susceptibility Testing (EUCAST; v13.0; 1 January 2023).

Isolates of penicillin MIC value ≤0.064 mg/L were categorised as ‘susceptible, standard dosing regimen (S)’ (where there is a high likelihood of therapeutic success using a standard dosing regimen of the agent) and isolates of penicillin MIC value >0.25 mg/L were categorised as ‘resistant (R)’ (where there is a high likelihood of therapeutic failure even when there is increased exposure). Isolates exhibiting intermediate penicillin MIC values 0.094–0.25 mg/L were categorised as ‘susceptible, increased exposure (I)’ (where there is a high likelihood of therapeutic success because exposure to the agent is increased by adjusting the dosing regimen or by its concentration at the site of infection) [40].

Isolates of ciprofloxacin MIC value ≤0.016 mg/L and rifampicin MIC value ≤0.25 mg/L were categorized as susceptible.

### 4.4. Multilocus Sequence Typing (MLST)

Isolates belonging to a serogroup were characterized by MLST typing, as described previously [41], using the *Neisseria* PubMLST database (http://pubmlst.org/neisseria/) (accessed on 20 February 2023) [26]. Sequence types (ST) were defined and grouped into clonal complexes (ccs).

### 4.5. Molecular Identification of penA, gyrA and rpoB Genes

A 402 bp fragment (*penA*) of the NEIS1753 (PBP2) gene was characterised by PCR by the use of penA1F and penA1R primers, as previously described by Taha et al. [15]. A 525 bp fragment of the Quinolone Resistance-Determining Region (QRDR) of *gyrA* gene was amplified by PCR, by the use of gyrA1F and gyrA1R primers, previously described by Hong et al. [42], while a 660 bp fragment of the *rpoB* gene, was characterized by PCR by the use of RpoB1F and RpoB1R primers, as previously described by Taha et al. [43] (Table S3).

### 4.6. Amplification Protocol

Amplification reactions contained 0.4 μM of each primer (VBC, Hamburg, Germany), 0.8 mM dNTPs (New England Biolabs, Ipswich, MA, USA), 0.5 U Phusion High-Fidelity DNA Polymerase (New England Biolabs), 1× reaction buffer GC, and 1 μL of DNA template in a total volume 25 μL. Polymerase chain reaction conditions were the following: 98 °C for 30 s; 98 °C for 20 s, 69 °C for 30 s, and 72 °C for 20 s (35 cycles); the final extension step was at 72 °C for 1 min (Robocycler Gradient 96 Cycler, Stratagene, La Jolla, CA, USA).

Positive and negative controls were included in each respective PCR assay. In particular, the *N. meningitidis* reference strains 4954, 4955, and 4956 were obtained from the UK NEQAS (in the frame of IBDLabNet External Quality Assurance scheme), which were used as positive controls, while PCR-grade water (Invitrogen^TM^, Life Technologies Corporation, Waltham, MA, USA) was used as a negative control.

Finally, gel electrophoresis was carried out in 10 μL of the PCR product stained with GelRed loading buffer (6× Gel loading dye, Biotium, Fremont, CA, USA) in 2% (*w*/*v*) agarose gel (Nippon Genetics, Tokyo, Japan) using a FastGene 100 bp DNA marker (MWD100, Nippon Genetics, Tokyo, Japan) and visualized under ultraviolet fluorescence light.

### 4.7. PCR Product Purification and Sequencing

PCR products were purified according to the PCR-clean-up protocol, NucleoSpin^®^ Gel, and a PCR clean-up kit (Macherey–Nagel, Düren, Germany) in a 30 μL final elution volume. Further, in order to test the purification yield, 5 μL of the purified product was stained with 1 μL GelRed loading buffer (6× Gel loading dye, Biotium, Fremont, CA, USA). Purified products were subjected to electrophoresis in 2.0% (*w*/*v*) agarose gel (Nippon Genetics, Tokyo, Japan) and visualized under ultraviolet fluorescence light. The purified products were sequenced by Sanger on an ABI 3730xl DNA analyzer (Applied Biosystems™, ThermoFisher Scientific, Waltham, MA, USA) using the BigDye™ Terminator v3.1 Cycle Sequencing Kit (Applied Biosystems™, ThermoFisher Scientific, Waltham, MA, USA).

### 4.8. Sequencing Analysis

Chromatograms were analyzed using Chromas software (http://technelysium.com.au/wp/chromas/, version 2.6.6, Technelysium Pty Ltd., South Brisbane, Australia, free downloaded) (accessed on 20 February 2023). Nucleotide sequences derived from the two DNA chains were compared to each other with ClustalW (https://www.genome.jp/tools-bin/clustalw/) (accessed on 20 February 2023) bioinformatics tools were provided by GenomeNet, Kyoto University Bioinformatics Center, Kyoto, Japan, free online software). Alleles at antigenic loci were assigned by the *Neisseria* PubMLST database (http://pubmlst.org/neisseria/) (accessed on 20 February 2023) [26].

## 5. Conclusions

In conclusion, we demonstrated that, during 12 years of IMD surveillance in Greece, resistance to ciprofloxacin and rifampicin remained rare. However, decreasing susceptibility was observed for penicillin. Phenotypic antimicrobial resistance surveys of isolates collected from Greek IMD cases, along with genetic investigations into the mechanisms of resistance, are important for ensuring that current antibiotic treatment and prophylaxis recommendations remain relevant.

## Figures and Tables

**Table 1:** Antibiotic susceptibility by year in Greece, 2010–2021.

	Number of Isolates by Year (%)												
MIC Values by Antibiotic	2010 (*n* = 22)	2011 (*n* = 16)	2012 (*n* = 17)	2013 (*n* = 18)	2014 (*n* = 21)	2015 (*n* = 18)	2016 (*n* = 26)	2017 (*n* = 17)	2018 (*n* = 14)	2019 (*n* = 15)	2020 (*n* = 6)	2021 (*n* = 2)	Total (*n* = 192)
Penicillin													
Pen^S^ ≤ 0.064 mg/L	16 (73%)	10 (63%)	13 (75%)	12 (67%)	10 (48%)	8 (45%)	12 (46%)	9 (53%)	2 (14%)	4 (27%)	2 (33%)	1 (50%)	99
Pen^I^ 0.094–0.25 mg/L	6 (27%)	6 (37%)	3 (19%)	5 (28%)	10 (48%)	6 (33%)	11 (42%)	6 (35%)	8 (57%)	9 (60%)	2 (33%)	0 (0%)	72
Pen^R^ > 0.25 mg/L	0 (0%)	0 (0%)	1 (6%)	1 (5%)	1 (4%)	4 (22%)	3 (12%)	2 (12%)	4 (29%)	2 (13%)	2 (33%)	1 (50%)	21
Ciprofloxacin													
Cip^S^ ≤ 0.016 mg/L	22	16	17	18	21	18	26	17	14	15	4	2	190
Cip^R^ > 0.016 mg/L	0	0	0	0	0	0	0	0	0	0	2	0	2
Rifampicin													
Rif^S^ ≤ 0.25 mg/L	22	16	17	18	21	17	26	17	14	15	6	2	191
Rif^R^ > 0.25 mg/L	0	0	0	0	0	1	0	0	0	0	0	0	1

## Data Availability

Data upon request.

## Supplementary Materials

The following supporting information can be downloaded at: https://www.mdpi.com/article/10.3390/antibiotics12071136/s1, Table S1: penA alleles among PenR and PenI meningococcal isolates in Greece, 2010 to 2021.; Table S2: Distribution of penA alleles in relation to serogroups and clonal complexes, Greece, 2010–2021.; Table S3: Oligonucleotide primers used for the PCR amplification and sequencing of *penA*, *gyrA* and *rpoB* genes.
