# Supplementary material for: Meningococcal Antibiotic Resistance: Molecular Characterization of Isolates from Patients with Invasive Meningococcal Disease (IMD) in Greece
Source: Antibiotics (Basel). 2023 Jun 30;12(7):1136. doi: 10.3390/antibiotics12071136 (PMC10376615; doi:10.3390/antibiotics12071136)
Supplement: Supplementary file 1 [file antibiotics-12-01136-s001.zip › antibiotics-2368084-supplementary.pdf]

Table S1. *penA* alleles among Pen^R and Pen^I meningococcal isolates in Greece, 2010-2021.

penA allele	Total number of isolates	Number of Pen^S isolates	Number of Pen^I isolates	Number of Pen^R isolates	MIC values of range (mg/L)	MIC_{50}	MIC_{90}	Number of penicillin resistance associated AAAs
1	20	19	1	0	0.016-0.125	0.047	0.064	0
2	2	1	1	0	0.047-0.125			0
3	35	25	10	0	0.016-0.19	0.064	0.125	0
4	2	1	1	0	0.064-0.094			0
5	4	4	0	0	0.023-0.047			0
7	1	0	1	0	0.25			5
9	6	0	0	6	0.38-0.5			5
10	3	0	2	1	0.25-0.5			5
12	3	0	3	0	0.125-0.25			5
14	13	2	11	0	0.025-0.25	0.125	0.19	5
15	1	0	1	0	0.25			5
19	2	0	2	0	0.25			5
20	1	0	1	0	0.19			5
21	2	0	1	1	0.25-0.38			5
22	13	7	6	0	0.032-0.094	0.064	0.094	0
23	1	1	0	0	0.064			5
25	9	0	7	2	0.094-0.38			5
27	35	33	2	0	0.006-0.094	0.032	0.064	0
29	1	0	1	0	0.125			5
33	1	0	1	0	0.19			5
34	4	3	1	0	0.012-0.094			0
38	1	1	0	0	0.032			0
52	1	0	1	0	0.094			5
77	1	1	0	0	0.064			5
79	1	0	1	0	0.19			5
154	1	1	0	0	0.064			0
184	1	0	1	0	0.25			0
248	4	0	4	0	0.25			5
295	8	0	2	6	0.25-0.5			5
420	1	0	0	1	0.38			5
426	2	0	1	1	0.25-0.5			5
567	1	0	1	0	0.094			5
568	1	0	1	0	0.25			5
569	3	0	3	0	0.19-0.25			5
693	1	0	1	0	0.19			5
694	1	0	1	0	0.25			5
695	1	0	1	0	0.19			5
910	2	0	0	2	0.5			5
1185	1	0	1	0	0.19			ND
1189	1	0	0	1	0.75			5

Alleles among 192 IMD isolates. Pen^S = penicillin-susceptible, standard exposure; Pen^I = Penicillin-susceptible, increased exposure; Pen^R = Penicillin-resistant according to EUCAST guidelines. MIC_{50} and MIC_{90} calculated for ⩾10 isolates. AASs = amino acid substitutions. ND = Not determined.

Table S2. Distribution of *penA* alleles in relation to serogroups and clonal complexes, Greece, 2010–2021.

			No of strains[a]																								
			MIC Pen			MenB (n=162)															MenY (n=12)		MenC (n=10)		MenW (n=7)			MenX (n=1)
penA allele	No of substitutions in PBP2	No of strains (n=192)	Pen[S] (n=99)	Pen[I] (n=72)	Pen[R] (n=21)	cc11 (n=1)	cc32 (n=27)	cc35 (n=8)	cc41/44 (n=28)	cc60 (n=6)	cc162 (n=11)	cc163 (n=1)	cc213 (n=16)	cc269 (n=41)	cc461 (n=2)	cc865 (n=5)	cc1136 (n=1)	cc1157 (n=1)	cc1572 (n=2)	UA (n=13)	cc22 (n=2)	cc23 (n=10)	cc11 (n=9)	cc41/44 (n=1)	cc11 (n=4)	cc22 (n=2)	cc167 (n=1)	UA (n=1)
3	0	35	25	10			26	4						1						3								1
27	0	35	33	2										35														
1	0	20	19	1					9	2	2									1			3		3			
5	0	4	4						3				1															
34	0	4	3	1									4															
23	5	1	1										1															
38	0	1	1									1																
77	5	1	1																		1							
154	0	1	1																	1								
2	0	2	1	1													1										1	
4	0	2	1	1		1														1								
22	0	13	7	6					3									1				9						
14	5	13	2	11					1	1	1			3						4	1					2		
25	5	9		7	2			1			8																	
10	5	3		2	1				2	1														1				
7	5	1		1																			1					
12	5	3		3									1						2									
15	5	1		1					1																			
19	5	2		2					2																			
20	5	1		1																		1						
29	5	1		1						1																		
33	5	1		1											1													
52	5	1		1			1																					
79	5	1		1																1								
184	0	1		1					1																			
248	5	4		4																			4					
567	5	1		1					1																			

568	5	1	1										1	
569	5	3	3			3								
693	5	1	1					1						
694	5	1	1						1					
695	5	1	1		1									
1185[b]	5	1	1						1					
9	5	6		6							5			1
910	5	2		2								2		
420	5	1		1						1				
1189[b]	5	1		1			1							
295	5	8	2	6				8						
21	5	2	1	1	2									
426	5	2	1	1		2								

[a]MenB, meningococcal of serogroup B; MenY, meningococcal of serogroup Y; MenC, meningococcal of serogroup C; MenW, meningococcal of serogroup W; MenX, meningococcal of serogroup X.

[b]New *penA* allele.

Table S3. Oligonucleotide primers used for the PCR amplification and sequencing of *penA*, *gyrA* and *rpoB* genes.

The universal forward and reverse sequences (shown in lowercase letters) were added as adapters to the 5′ end upstream and downstream from the oligonucleotides and were then used for sequencing.

Gene	Primers	Sequences (5′ – 3′)	Amplicon Size (bp)	Publication
penA	penA1F	gttttcccagtcacgacgttgtaATCGAACAGGCGACGATGTC	402	Taha 2007 [15]
	penA1R	ttgtgagcggataacaatttcGATTAAGACGGTGTTTTGACGG		
gyrA	gyrA1F	gttttcccagtcacgacgttgtaATGACCGACGCAACCATCCGCCAC	525	Hong 2013 [42]
	gyrA1R	ttgtgagcggataacaatttcCCAGCTTGGCTTTGTTGACCTGATAG		
rpoB	rpoB1F	gttttcccagtcacgacgttgtaCTGTCCGAAGCCCAACAAAACTCTTGG	660	Taha 2010 [43]
	rpoB1R	ttgtgagcggataacaatttcTTCCAAGAATGGAATCAGGGATGCTGC		
